# Interleukin 32γ (IL-32γ) is highly expressed in cutaneous and mucosal lesions of American Tegumentary Leishmaniasis patients: association with tumor necrosis factor (TNF) and IL-10

**DOI:** 10.1186/1471-2334-14-249

**Published:** 2014-05-09

**Authors:** Hélio Galdino, Anetícia Eduarda Maldaner, Lívia Lara Pessoni, Frederico M Soriani, Ledice Inácia de Araújo Pereira, Sebastião Alves Pinto, Fernanda Bugalho Duarte, Clayson Moura Gomes, Anna Karoline Aguiar Fleuri, Miriam Leandro Dorta, Milton Adriano Pelli de Oliveira, Mauro Martins Teixeira, Aline Carvalho Batista, Leo A B Joosten, Leda Quercia Vieira, Fátima Ribeiro-Dias

**Affiliations:** 1 Institute of Tropical Pathology and Public Healthy, Universidade Federal de Goiás, Rua 235 S/N – Setor Universitário, Goiânia 74605-050, Goiás, Brazil; 2 Department of Biochemistry and Immunology, Universidade Federal de Minas Gerais, Belo Horizonte, Minas Gerais, Brazil; 3 Department of Pathology, Faculty of Medicine, Universidade Federal de Goiás, and Instituto Goiano de Oncologia e Hematologia (INGOH), Goiânia, Goiás, Brazil; 4 Otorhinolaryngology, Hospital Unique, Goiânia, Goiás, Brazil; 5 Department of Pathology, Faculty of Odontology, Universidade Federal de Goiás, Goiânia, Goiás, Brazil; 6 Department of Internal Medicine, Radboud University Nijmegen Medical Centre, Nijmegen, The Netherlands

**Keywords:** IL-32, Leishmaniasis, TNF, IL-10, *Leishmania* (*Viannia*) sp

## Abstract

**Background:**

The interleukin 32 (IL-32) is a proinflammatory cytokine produced by immune and non-immune cells. It can be induced during bacterial and viral infections, but its production was never investigated in protozoan infections. American Tegumentary Leishmaniasis (ATL) is caused by *Leishmania* protozoan leading to cutaneous, nasal or oral lesions. The aim of this study was to evaluate the expression of IL-32 in cutaneous and mucosal lesions as well as in peripheral blood mononuclear cells (PBMC) exposed to *Leishmania* (*Viannia*) *braziliensis*.

**Methods:**

IL-32, tumour necrosis factor (TNF) and IL-10 protein expression was evaluated by immunohistochemistry in cutaneous, mucosal lesions and compared to healthy specimens. The isoforms of IL-32α, β, δ, γ mRNA, TNF mRNA and IL-10 mRNA were assessed by qPCR in tissue biopsies of lesions and healthy skin and mucosa. In addition, PBMC from healthy donors were cultured with amastigotes of *L.* (*V.*) *braziliensis*. In lesions, the parasite subgenus was identified by PCR-RFLP.

**Results:**

We showed that the mRNA expression of IL-32, in particular IL-32γ was similarly up-regulated in lesions of cutaneous (CL) or mucosal (ML) leishmaniasis patients. IL-32 protein was produced by epithelial, endothelial, mononuclear cells and giant cells. The IL-32 protein expression was associated with TNF in ML but not in CL. IL-32 was not associated with IL-10 in both CL and ML. Expression of TNF mRNA was higher in ML than in CL lesions, however levels of IL-10 mRNA were similar in both clinical forms. In all lesions in which the parasite was detected, *L*. (*Viannia*) subgenus was identified. Interestingly, *L*. (*V*.) *braziliensis* induced only IL-32γ mRNA expression in PBMC from healthy individuals.

**Conclusions:**

These data suggest that IL-32 plays a major role in the inflammatory process caused by *L*. (*Viannia*) sp or that IL-32 is crucial for controlling the *L*. (*Viannia*) sp infection.

## Background

The interleukin 32 (IL-32) was initially described as natural killer (NK) cell transcript 4 (NK4) expressed in human activated NK and T cells [1]. After the description of several inflammatory cytokine properties of the NK4 this protein was named IL-32 [2]. The four main isoforms of IL-32 (α, β, γ, δ), regulated by mRNA alternative splicing, are expressed in both immune and nonimmune cells. Two other isoforms of IL-32 (ϵ, ζ) that were described shortly thereafter are highly expressed in activated T lymphocytes [2,3]. The isoform IL-32γ presents the highest biological activity [4]. Sequence homology between IL-32 and other cytokines was not found, and until now a homologous gene in mouse is not identified [2]. Most of IL-32 is cell-associated, however depending on the cell type and stimulus it can be released [2,5-7]. IL-32 is expressed in inflammatory and infectious diseases as well as in healthy tissue, mostly in epithelial cells [7-9]. Primary keratinocytes produces IL-32 after activation with interferon gamma (IFNγ) and tumour necrosis factor (TNF) [7]. In addition, TNF, IFNγ, and IL-1β induce IL-32 in intestinal epithelial cell lines [10]. Immune cells, such as NK cells stimulated with IL-12/IL-18, and peripheral blood mononuclear cells (PBMC) cultured with mitogens also express IL-32 [2]. Monocytes/macrophages and dendritic cells express IL-32 upon toll-like receptor (TLR) activation [5,11].

During inflammation, IL-32 can be induced by inflammatory cytokines, and, in turn, IL-32 also induces other inflammatory mediators. It has been demonstrated that recombinant IL-32 (α and β) induces TNF and IL-8 in human monocyte-like THP-1 cells and in murine macrophage cell line RAW 264.7, as well as CXCL2 chemokine in murine primary macrophages. The induction of cytokines by IL-32 involves activation of transcription factor NFκB and p38 mitogen-activated protein kinase [2]. Moreover, IL-32 induces inflammatory mediators (TNF, IL-6, prostaglandin E_2_) as well as anti-inflammatory cytokine (IL-10) in human monocytes and monocyte-derived dendritic cells [11,12]. IL-32γ synergizes with nucleotide oligomerization domain 1 and 2 (NOD1/NOD2) ligands and with TLR2 agonist to induce IL-1β [9,13]. Such effects has pointed out IL-32 as an important player in the pathogenesis of inflammatory diseases as atopic dermatitis, Crohn´s disease, rheumatoid arthritis and rhinitis [7,10,12,14]. Especially a cross-talk between IL-32 and TNF has been described to be relevant in the pathogenesis of these diseases [15-17]. During infection caused by influenza A virus [18], human immunodeficiency virus [19] or *Mycobacterium avium*[8] an increase of IL-32 production has been reported. *Mycobacterium tuberculosis* can induce IL-32 in human monocytes in a caspase-1/IL-18/IFNγ-dependent manner [5]. Microbicidal mechanisms can be induced by IL-32 during these infections [18,20]. Together, these data indicate that microorganisms can induce IL-32 expression in immune and nonimmune cells to control the infection, but alternatively this cytokine might play a role in the pathogenesis of the diseases.

American Tegumentary Leishmaniasis (ATL) is a chronic inflammatory disease caused by *Leishmania* parasites*.* In Brazil, the species that belong to *Leishmania* (*Viannia)* subgenus can induce different clinical manifestations of the disease. The localized cutaneous leishmaniasis (CL) is most frequent and patients can present spontaneous clinical cure. However, mucosal leishmaniasis (ML) which affects nasopharyngeal and oral mucosal (with ulceration and septum perforation) never heals spontaneously, it is difficult to treat, and recurrence is frequent [21,22]. ML can occur simultaneously with cutaneous lesions (mucocutaneous leishmaniasis), however, most of the ML cases occur after cutaneous lesions (months or years) or in the absence of any signal of previous CL [22-25]. The detection of ML concomitantly with cutaneous lesions is a rare event (~2.7% of ML) [26].

In ATL, a cellular immune response (Th1 lymphocytes) is crucial to control the infection, but it is also deleterious to tissues leading to ulcerated lesions. The Montenegro´s skin test (delayed hypersensitivity) reveals the strong Th1 response in ATL patients [23]. Typical Th1 cytokines, IFNγ and TNF, are detected in lesions and these cytokines promote macrophage activation to control the parasite [27] but they also promote inflammation and tissue destruction [23,28,29]. Besides inflammatory cytokines, IL-10 is also detected in ATL lesions [28,29]. A balance between TNF/IFNγ and IL-10 seems to be important to control the infection and the inflammatory process [30,31]. Because these cytokines are related to the production and biological function of IL-32 we hypothesized that IL-32 can be induced during *Leishmania* infection and to contribute to the pathogenesis of the disease. Until now, the presence of IL-32 was not evaluated in leishmaniasis and in ATL IL-32 could be a crucial mediator between epithelium and inflammatory cells. As it has been demonstrated that IL-32γ can be spliced to IL-32β, which is less inflammatory suggesting a balance between IL-32γ/IL-32β to control inflammation [32], we also evaluated which isoforms of IL-32 are present during ATL.

## Methods

### Patients, controls, and tissue samples

In Annuar Auad Tropical Disease Hospital, in Goiânia, Goiás, West Central region of Brazil, patients were diagnosed with cutaneous (n = 24) or mucosal (n = 10) ATL. To be included in the study, besides a typical leishmaniasis lesion, patients presented at least one exam indicating the presence of parasite (Polymerase chain reaction, PCR for *Leishmania* genus; histopathological analysis with immunohistochemistry (IHC) for amastigotes); and also Indirect immunofluorescence (IFI) and Montenegro´s skin test (MST) were used at the diagnosis. All technical procedures were described in Oliveira et al. [33]. Patients did not perform all tests. Before treatment, biopsy specimens were obtained from cutaneous or nasal/oralpharyngix lesions of ATL patients, and from healthy tissues during plastic surgery (for cutaneous tissue, n = 8) or tonsil or nasal septum surgeries (for mucosal tissues, n = 7). One fragment of patient biopsy was fixed in 10% buffered formalin and embedded in paraffin for histological and IHC analysis; another one was stored in TRIzol reagent (Invitrogen, Carlsbad, CA, USA) for gene expression evaluation, and the last one was stored in saline (-20°C) for molecular characterization of parasites. For some patients the amount of tissue fragment was not enough for all analyses. From another set of healthy donors (n = 21, a blood sample was collected (5 ml) for PBMC isolation. All procedures were approved by Ethics Committee in Research of Universidade Federal de Goiás (protocol number 165/08) and informed consent was signed by all patients and controls (the study included only individuals with age > 18 years).

### Molecular characterization of *Leishmania*

First, the PCR was done to detect *Leishmania* genus (for diagnosis of leishmaniasis), then the polymerase chain reaction-restriction fragment length polymorphism (PCR-RFLP) was used to identify *Leishmania* subgenus in biopsy fragments. Briefly, tissue lysates were obtained from frozen biopsy fragments by adding 25 μl of TE (10 mM Tris–HCl, 1 mM EDTA, pH 8.0), and proteinase K (100 μg/ml), at 56°C, for 3 h. The DNA was extracted using the *illustra™–blood genomicPrep Mini Spin Kit* (GE Healthcare UK Limited, Buckinghamshire, UK) as recommended by the manufacturer. The PCR was carried out using 1 μM of each primer (to conserved region of minicircles of kDNA), 150: 5- GGG(G/T)AGGGGCGTTCT(C/G)CGAA 3- and 152: 5- (C/G)(C/G)(C/G)(A/T)CTAT(A/T) TTACACCAACCCC 3- [34], together with 200 μM of dNTPs, including dUTP instead of dTTP, 0.8 U of Taq DNA polymerase (Invitrogen), buffer (10 mM Tris–HCl pH 8.6, 50 mM KCl, 1.5 mM MgCl2) and 1 μl of DNA template, in a final volume of 10 μl. Amplification was carried out in Eppendorf PTC-100 machine, using an initial denaturation step at 94°C for 5 min, followed by 29 cycles at 94°C for 1 min, 59°C for 45 s, 72°C for 30 s and a final extension step of 7 min. The PCR products were run in an 8% polyacrylamide gel in a Mini-Protean II apparatus (Bio-Rad, Hercules, CA, USA), followed by silver staining to visualize the bands. Positive controls containing DNA of *L*. (*V*.) *braziliensis* or *L*. (*L*.) *amazonensis* and a negative control without DNA were included in each reaction set. All positive samples were subjected to RFLP analysis of the amplification product [35]. The PCR products (5 μL) were used for digestion by the addition of 1 U of *Hae* III (Invitrogen) for 3 h, at 37°C. The restriction fragments were separated in a 10% polyacrylamide gel in the Mini-Protean II apparatus (Bio-Rad) and silver stained. The *Hae* III digested the 120 bp PCR product of *L*. (*V*.) *braziliensis* producing two fragments, one of 40 bp and other of 80 bp and the enzyme did not digest *L*. (*L*.) *amazonensis* amplicon.

### Immunohistochemical (IHC) analysis for IL-32, IL-10, TNF

Paraffin-embedded tissues were sectioned (3 μm) and collected in series on glass slides coated with 2% 3-aminopropyltriethsilane (Sigma Aldrich, St. Louis, MO, USA). The sections were deparaffinised by immersion in xylene, and then were immersed in alcohol. For antigen retrieval, the sections were immersed in 10 mM Tris/1 mM EDTA buffer pH 9.0, for 30 min at 95°C, and then incubated with 3% hydrogen peroxide (Merck SA, RJ, Brazil) for 40 min. Sections were blocked by incubation with Background Sniper (blocked protein, Starr Trek Universal HRP Detection System, Biocare Medical Inc., Concord, CA, USA) for 15 min. The sections were immediately incubated with the following primary rabbit polyclonal antibodies (1:100 dilution): anti-IL32 (Abcam Inc., Cambridge, MA, USA), anti-IL10 or anti-TNF (Abcam Inc.), at 4°C, overnight, in a humidified chamber. After washing in Tris-Buffered Saline, the sections were treated with Link Universal Trekie (Starr Trek Universal HRP Detection System), for 20 min, followed by washing and treatment with TrekAvidin-HRP (Starr Trek Universal HRP Detection System) for 20 min. Sections were then incubated in Betazoid DAB Chromogen solution (Biocare Medical Inc.) and counterstained with Mayer’s haematoxylin for 30 s. Negative controls were obtained by omission of the primary antibodies, which were substituted by 1% phosphate-buffered saline (PBS) – Bovine Serum Albumin (Sigma). Sections were coded and blindly analysed by two observers. Expression of cytokines was semi quantitatively scored under light microscopy (400× magnification, 10 fields per section), and classified according scores: 0 (absence of positive cells), 1 (1% to 25% of positive cells), 2 (26% to 50% of positive cells), 3 (51% to 75% of positive cells) and 4 (76% to 100% of positive cells), adapted from Joosten *et al.*[12]. The data were represented as individuals and median values.

### Cultures of PBMC and amastigote forms of *Leishmania* (*V.*) *braziliensis*

Venous blood was collected in tubes containing EDTA (BD Vacutainer^TM^, Juiz de Fora, MG, Brazil), centrifuged (240 *g*, 15 min, room temperature) and plasma was discarded. The blood was diluted 1:2 in 0.01 M EDTA-PBS pH 7.3 and layered on Ficoll (Ficoll Paque Plus, GE Healthcare). After centrifugation (1.400 *g*, 15 min, room temperature), PBMC were collected, washed twice in PBS (600 *g*, 10 min, 4°C) and suspended in RPMI 1640 medium (Sigma) supplemented with 10% inactivated fetal calf serum (FCS, Gibco), 2 mM L-glutamine (Sigma), 100 U/ml penicillin (Sigma), and 100 μg/ml streptomycin (Sigma). The isolate *L. (V.) braziliensis* MHOM/BR/2003/IMG^*Lb*^, from *Leishbank* (West Central region *Leishmania* bank, Universidade Federal de Goiás, Brazil), characterized as previously described [36], was used to generate amastigote forms based on Ahmed *et al.*[37] and Lang *et al.*[38] with some modifications. Briefly, promastigote forms were cultured in 24-well plates in Grace`s insect culture medium (Sigma) supplemented with 20% heat-inactivated FCS (Cripion, Andradina, Brazil), 2 mM L-glutamine, 100 U/ml penicillin, and 100 μg/ml streptomycin, at 26°C (6 days). The parasites were washed with PBS and injected subcutaneously (5 × 10^6^/50 μl) into one of the hind footpads of IFNγ-deficient mice as previously described [33]. When lesion size was around 2 - 3 mm, animals were sacrificed by CO_2_ inhalation and the footpads were aseptically collected, macerated in PBS, and layered on Percoll (GE Healthcare; 44% and 100%, 2.500 g, 30 min, 10°C) to obtain amastigotes. The PBMC (1.2 × 10^6^/400 μl) were cultured with amastigotes (3 × 10^5^/400 μl) in duplicates, at 36°C, 5% CO_2_. After 24 h, PBMC were stored (RNA Store, DNR, Jerusalem, Israel) for further IL-32 investigation.

### Quantitative reverse transcription-polymerase chain reaction (qPCR) for IL-32 isoforms, TNF and IL-10

From normal tissues, cutaneous or mucosal lesions and PBMC, RNA extraction was done by Trizol method (Invitrogen). The cDNA was synthetized using SuperScript III reverse transcriptase (Invitrogen) and Real-Time PCR reactions were done in an Applied Biosystems 7500 FAST device using the IL-32 primers designed by Heinhuis *et al.*[32], and detected by SYBR Green Mastermix (Applied Biosystems, Foster City, CA, USA). Primer sequences are presented in Table 1. Briefly, the reactions were done in a 10 μl final volume containing 5 μl of SYBR Green Mastermix, 1 μl of each primer (5 μM), 2 μl of deionised water, and 1 μl of cDNA. The reactions were initialized with 60°C for 20 s, followed by an step of 95°C for 10 min, 40 cycles at 95°C for 10 s, and 60°C for 1 min. At the end of the reactions it was performed a melting curve. GAPDH was used as the reference housekeeping gene. The results were analysed using the 2^-ΔΔCt^ method.

**Table 1:** Sequences of primers

**Cytokine**	**Sense**	**Anti-sense**
IL-32α	5′GCTGGAGGACGACTTCAAAGA3′	5′GGGCTCCGTAGGACTTGTCA3′
IL-32β	5′CAGTGGAGCTGGGTCATCTCA3′	5′GGGCCTTCAGCTTCTTCATGTCATCA3′
IL-32γ	5′AGGCCCGAATGGTAATGCT3′	5′CCACAGTGTCCTCAGTGTCACA3′
IL-32δ	5′TCTGTCTCTCTCGGGTCCTCTCT3′	5′TGTCTCCAGGTAGCCCTCTTTG3′
IL-10	5′GGTTCGCAAGCCTTGTCTGA 3′	5′TCCCCCAGGGAGTTCACAT 3′
TNF	5′AAGCCTGTAGCCCATGTTGT 3′	5′CAGATAGATGGGCTCATACC 3′
GAPDH	5′CTCAAGATTGTCAGCAATGC 3′	5′ CAGGATGCCCTTTAGTGGGC 3′

Sequences of primers used to detect cytokine mRNA by using qPCR in leishmaniasis lesions and control tissues.

### Statistical analyses

Data represent mean ± standard deviation (SD), individual values, medians [minimal and maximal values]. Mann Whitney and Spearman´s correlation tests were performed using GraphPad Prism 5.0 Software Inc. (San Diego, CA, USA). *p* < 0.05 was considered significant.

## Results

### Characterization of patients and parasites

As presented in Table 2, 24 patients with CL and 10 with ML were evaluated. The mean of patient age was 38.5 ± 15.1 years for CL and 57.2 ± 17.9 years for ML patients. Most of patients were infected in Central Brazil (67.6%, from Goiás and Mato Grosso), but there were cases from North and Northeast Brazilian regions. In CL, the number of lesions was 3.5 ± 5.0, and lesion sizes were 2.7 ± 2.2 cm. The duration of the disease was higher in ML than in CL, varying from 15 days until 48 months in CL and from two months until 360 months in ML group. ML patients only presented mucosal lesions, but seven patients reported history of previous cutaneous lesions. The majority of ML patients showed nasal lesions (9/10) with or not oralpharyngix involvement (3/9). All patients presented at least one parasitological positive test. At the diagnosis, patients were tested for *in vivo* immune cellular response, varying the MST result from 5.0 until 35 mm in CL patients, whereas in ML patients it varied from 7 until 50 mm. In CL group, half of patients presented two positive exams for presence of parasites (PCR and IHC for amastigotes, 12/24, 50.0%), and most of them was positive MST and positive PCR (14/24, 58.3%), while a less number of positive MST and positive IHC (9/24, 37.5%) was detected. In ML group, most of patients presented positive MST and positive PCR and/or IHC (8/10, 80.0%). The clinical and histopathological examinations of the lesions revealed that all were ulcerated presenting an intense inflammatory cell infiltration composed by macrophages and lymphocytes and showed a low number of parasites. These cells could be or not arranged in granulomas containing giant cells.

**Table 2:** **Characteristics of ATL patients**^**a**^

	^**b**^**CL**	^**c**^**ML**
**Male**	19/24	6/10
**Female**	5/24	4/10
**Age (years)**	38.5 ± 15.1	57.2 ± 17.9
**Number of lesions**	3.5 ± 5.0	_
**Size of Lesions (cm)**	2.7 ± 2.2	_
**Time of lesions (months)**	7.2 ± 10.2	117.0 ± 51.0
^**d**^**MST (mm)**	16.1 ± 7.6	21.4 ± 13.8
**Positive diagnostic test results% (positive/total tested)**		
^**e**^**PCR**	83.3 (20/24)	87.5 (7/8)
^**f**^**IHC**	69.6 (16/23)	66.6 (6/9)
^**g**^**IFI**	41.2 (7/17)	50.0 (4/8)
**MST**	76.2 (16/21)	100 (8/8)

^a^Thirty four ATL patients who were assisted at Hospital de Doenças Tropicais Anuar Auad (Goiânia - Goiás, West Central, Brazil), 24 with ^b^CL (Cutaneous Leishmaniasis) and 10 with ^c^ML (mucosal leishmaniasis). ^d^MST: Montenegro skin test; ^e^PCR: polymerase chain reaction for *Leishmania* genus; ^f^IHC: histopathological exam with immunohistochemistry for amastigotes; ^g^IFI: indirect immunofluorescence; 21 patients were from West Central region of Brazil (including 9 with ML; one was from South East); 10 from North and one from North East.

After PCR, there was amplification of the *Leishmania* kDNA in 27 samples (84.4%, 27/32), being 83.3% CL (20/24) and 87.5% ML (7/8); in two patients the PCR was not performed). In these positive samples all parasites belonged to *L*. (*Viannia*) subgenus.

### Expression of IL-32 protein is increased in cutaneous and mucosal lesions of ATL patients

**Figure 1:**
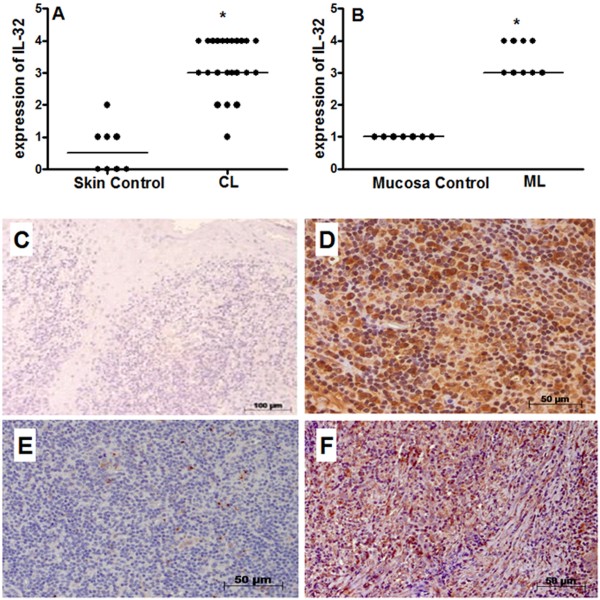
**IL-32 is detected in cutaneous and mucosal lesions of ATL patients.** Fragments of cutaneous lesions (CL, n = 23, **A**) or mucosal lesions (ML, n = 9, **B**) and healthy tissues (skin, n = 8; mucosa, n = 7) were included in paraffin and sections were submitted to IHC for IL-32. After reaction, the expression of IL-32 was determined through positive cells, analysed under light microscopy. The data represent individuals and medians values. *p < 0.05. Panel **C**: photomicrography of skin control. Panel **D**: photomicrography of CL lesion positive for IL-32. Panel **E**: photomicrography of mucosa control. Panel **F**: photomicrography of ML lesion positive for IL-32.

**Figure 2:**
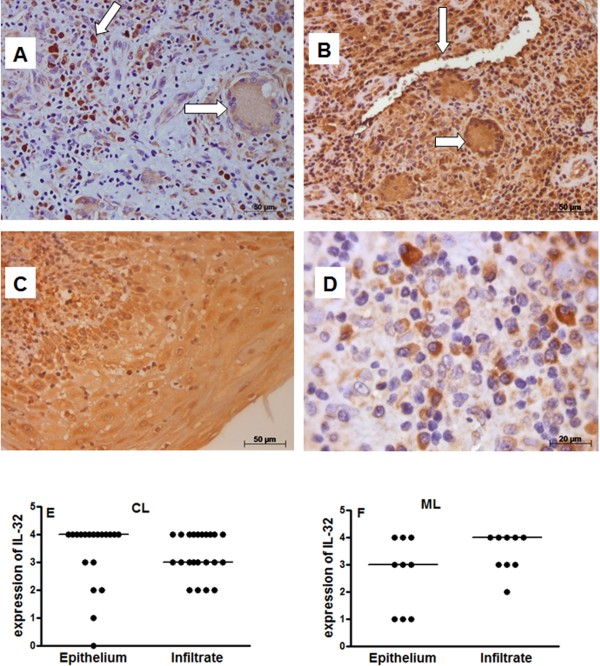
**Expression of IL-32 is detected in different cells.** Fragments of cutaneous or mucosal lesions were included in paraffin and sections were submitted to IHC for IL-32. The arrows show positive cells: In panel **A**: mononuclear cell and giant cell; and Panel **B**: endothelial cells and giant cell. In Panel **C**: positive keratinocytes; and Panel **D**: several positive mononuclear cells. The expression of IL-32 was determined, under light microscopy, in epithelium and infiltrate of CL (**E**, n = 22) and ML (**F**, n = 9). The data represent individual and median values.

The expression of IL-32 was increased in cutaneous lesions (CL: 3 [1 - 4] *vs.* Controls: 0.5 [0 - 2], *p* < 0.05, Figure 1A, 1D) as well as in mucosal lesions (ML: 3 [3 - 4] *vs.* Controls: 1 [1 - 1], *p* < 0.05, Figure 1B, 1F). Similar distribution of IL-32 was found in cutaneous or mucosal lesions. As IL-32 can be produced by epithelial cells and mononuclear cells, IL-32 expression was independently determined in the epithelium and inflammatory infiltrate. The IL-32 was found in several cells such as giant cells, endothelial cells, mononuclear cells and keratinocytes/epithelial cells (Figure 2A–D). The expression of IL-32 in cutaneous and mucosal lesions was similarly detected in the epithelium and in the inflammatory infiltrate (Figure 2E, 2F). Comparing the expression of IL-32 detected in the epithelium of cutaneous and mucosal lesions, no significant differences were found. Similar results were also obtained considering the infiltrate.

### The isoform IL-32γ is up-regulated in ATL lesions

**Figure 3:**
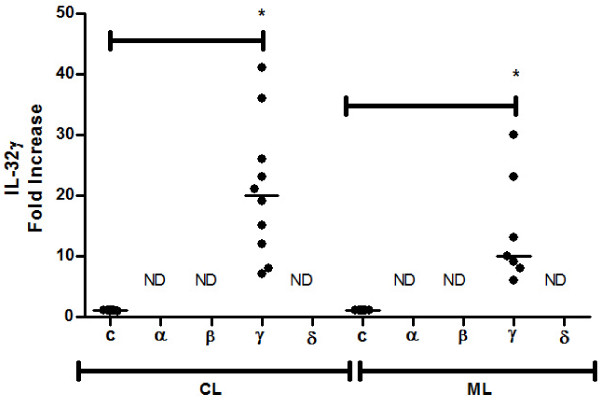
**IL-32γ mRNA is detected in lesions of patients with cutaneous (CL) and mucosal (ML) leishmaniasis.** Total mRNA was extracted from fragments of healthy tissues (C, skin n = 8; mucosa n = 7), cutaneous lesions (CL, n = 10) or mucosal lesions (ML, n = 7), and analysed by qPCR. Expression of isoforms α, β, γ and δ of IL-32 were analysed but only γIL-32 mRNA was detected. ND = Not detected. Results shown are individual values and medians. *p < 005.

As there are different isoforms of IL-32, we investigated the levels of mRNA of the IL-32 isoforms in healthy tissues and in cutaneous and mucosal lesions. Among the IL-32 isoforms investigated (α, β, γ, δ), only IL-32γ mRNA was detected. Similar to the protein expression, significantly higher levels of mRNA IL-32γ was detected in cutaneous and mucosal lesions than in healthy tissues. The isoform IL-32γ presented 20-fold increase in cutaneous lesions, and 10-fold increase in mucosal lesions in relation to control tissues. No significant differences were found when CL was compared with ML (Figure 3).

### Association of IL-32 expression with TNF and IL-10

**Figure 4:**
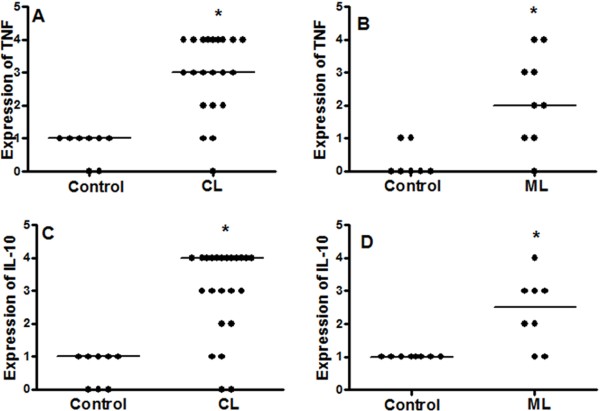
**Expression of TNF and IL-10 is increased in cutaneous and mucosal lesions of ATL patients.** Fragments of cutaneous **(CL, A and C)**, mucosal **(ML, B and D)**, and healthy tissues (Control) were included in paraffin and sections were submitted to IHC for TNF **(A and B)** and IL-10 **(C and D)**. After reaction, the expression of cytokines was determined through quantification of positive cells, under light microscopy (400×). Data represent individual and median values. TNF CL, n = 21; Control CL, n = 8. IL-10 CL, n = 23; Control CL, n = 8. TNF ML, n = 9; Control ML, n = 7. IL-10 ML and Control, n = 8. *p* < 0.05.

To evaluate the association between IL-32 and other cytokines in ATL, lesions and healthy tissues were submitted to IHC for detecting TNF and IL-10. These cytokines were variably expressed in cutaneous (CL: TNF = 3 [0 - 4] *vs.* Controls: 1 [0– 1], *p* < 0.05, Figure 4A; IL-10 = 4 [0 - 4] *vs.* Controls: 1 [0 - 1], *p* < 0.05, Figure 4C) as well as in mucosal lesions (ML: TNF = 2 [ 0 - 4] *vs.* Controls: 0 [0 – 1], *p* < 0.05, Figure 4B; IL-10 = 2.5 [1 - 4] *vs.* Controls 1 [1 - 1], *p* < 0.05; Figure 4D). No differences were detected in levels of TNF or IL-10 between CL and ML lesions. Concerning the correlations between cytokine expressions, IL-32 levels were not correlated with TNF and IL-10 levels in CL; however, in ML a positive correlation between IL-32 and TNF was detected (r = 0.88, n = 9, p < 0.05), but not between IL-32 and IL-10 (Table 3). A tendency of positive correlation was found between expression of TNF and IL-10 in CL group (r = 0.10, n = 20, Table 3).

**Table 3:** **Correlations between cytokines expressed in cutaneous and mucosal lesions detected as protein and mRNA**^**a**^

	**CL**^**b**^			**ML**^**c**^		
**Correlations**	**n**	**r**	**p**	**n**	**r**	**p**
IL-32 x TNF	21	-0.15	0.51	9	0.88	0.003*
IL-32 x IL-10	22	-0.03	0.90	8	0.11	0.79
TNF x IL-10	20	0.38	0.10	8	0.38	0.36
mRNA IL-32 *vs.* mRNA TNF	8	-0.17	0.70	7	-0.21	0.66
mRNA IL-32 *vs.* mRNA IL-10	8	-0.17	0.70	7	0.18	0.71
mRNA TNF *vs.* mRNA IL-10	10	0.28	0.43	6	0.65	0.18

^a^IL-32, TNF and IL-10 proteins were detected by IHC and IL-32γ, TNF and IL-10 mRNA were detected by qPCR, in biopsy fragments of lesions from ^b^Cutaneous leishmaniasis (CL) and ^c^Mucosal leishmaniasis (ML) patients. n = number of samples; r = Spearman´s correlation test. *p < 0.05.

**Figure 5:**
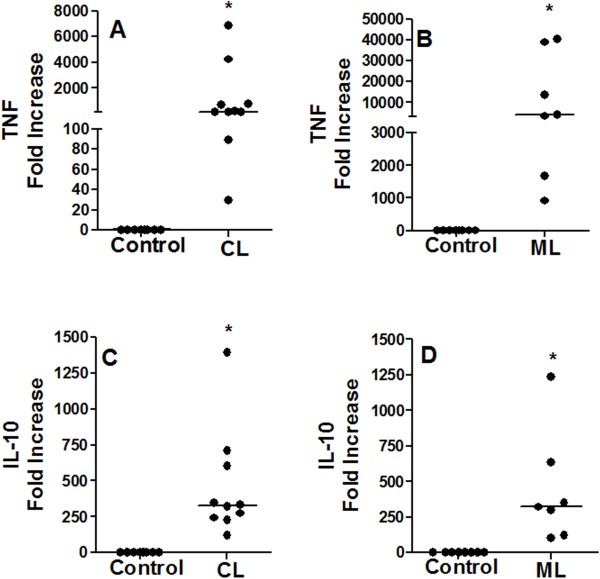
**Increase of TNF mRNA and IL-10 mRNA in lesions of ATL patients: high levels of TNF mRNA in mucosal leishmaniasis.** Total mRNA was extracted from fragments of healthy tissues (Control, skin n = 8; mucosa n = 8), cutaneous (CL, **A** and **C**, n = 10) or mucosal (ML, **B** and **D**, n = 7) lesions, and the expressions of TNF mRNA **(A, B)** and IL-10 **(C, D)** were analysed by qPCR. Results are showed as individual and median value. *p < 0.05.

To confirm and quantify the TNF and IL-10 expression in tissues, a qPCR was performed. The levels of TNF mRNA were higher in ML than in CL lesions (ML: 4,045.0 [924.0 - 40,307.0]; CL: 163.5 [29.8 - 6,888.0], Figure 5A and 5B). However, similar to the IHC results, the levels of IL-10 mRNA were similar in cutaneous and mucosal lesions (CL: 328.5 [120.0 - 1,395.0]; ML: 321.0 [103.0 - 1,237.0], Figure 5C e 5D). No associations between levels of TNF or IL-10 mRNA with IL-32 mRNA were detected (Table 3). It was observed a negative correlation between TNF mRNA and protein levels in ML group (r = -0.91, p < 0.05, n = 6).

### Amastigotes of *L*. (*V*.) *braziliensis* induces IL-32γ in mononuclear cells from health donors

**Figure 6:**
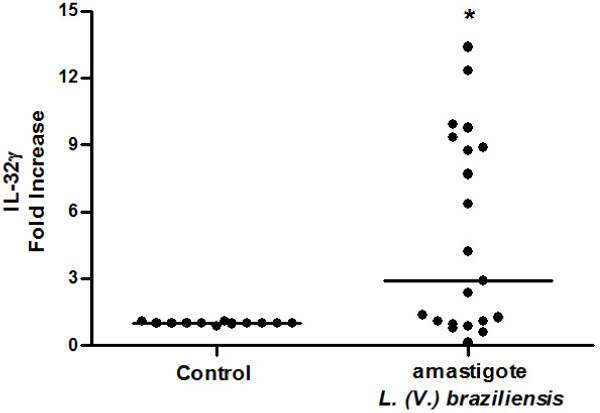
***L*. (*V*.) *braziliensis *induces IL-32γ mRNA in human mononuclear cells.** Mononuclear cells (3 × 10^6^/mL) from healthy blood donors were incubated with amastigote forms of *L*. (*V*.) *braziliensis* (3 × 10^5^/well) for 24 h. The IL-32γ mRNA was analysed by qPCR. Data represent individual values and medians (n = 21, *p < 0.05).

To evaluate whether the protozoan *Leishmania* was able to induce IL-32, PBMC from healthy donors were incubated with amastigotes of *L*. (*V*.) *braziliensis* for 24 h. By using qPCR four isoforms of IL-32 (α, β, γ, δ) were investigated, but interestingly only the IL-32γ mRNA was detected in PBMC cultures. There was ~3-fold increase in IL-32γ in cells incubated with amastigotes in comparison to cultures without parasites (Figure 6).

## Discussion

In the present study it was demonstrated for the first time that IL-32 is produced during *Leishmania* infection. Patients presenting cutaneous or mucosal forms of ATL produced similar levels of IL-32 protein and mRNA in their lesions. In ATL lesions, the expression of IL-32 was detected in several types of cells, including epithelial, endothelial and mononuclear cells. The IL-32 expression in different cell types has been previously described. In lung of *M. avium* complex-infected patients IL-32 was detected in alveolar macrophages and epithelial cells [8]. The presence of IL-32 in endothelial cells was demonstrated indicating a role for this cytokine in inflammation [39]. In keratinocytes and other epithelial cells, *in vitro* or *in vivo*, during infections or inflammatory diseases IL-32 is produced [7,8,10,40-42]. In the present study, IL-32 was detected in giant cells in some patient lesions presenting tuberculoid granuloma. Recently, Schenk *et al.*[43] showed that IL-32 is expressed in granulomas in leprosy lesions with a higher frequency of IL-32^+^ cells in tuberculoid than in lepromatous lesions. However, the authors did not describe whether giant cells were positive or not for IL-32. In leprosy, Th1 or Th2 lymphocyte responses are associated with tuberculoid and lepromatous leprosy, respectively. However, in ATL this polarization is not so clear, being detected Th1 as well as Th2 cytokines in cutaneous or mucosal lesions with a predominance of Th1 cytokines [23,28,44]. In some ATL lesions it is possible to detect tuberculoid granuloma [23,45]. The presence of IL-32 in giant cells in ATL granuloma suggests the involvement of this cytokine in tuberculoid granuloma that deserves further investigation.

In this study, only IL-32γ isoform was detected in CL or ML lesions by using qPCR. The isoforms IL-32γ and IL-32α are mainly produced by mononuclear cells after activation [5] and they are detected in nasal mucosa tissue during allergic rhinitis [14]. The IL-32α is also detected in epithelial cells from colon mucosa during inflammatory bowel disease [9,10] and IL-32β is expressed in endothelial cells [39] and also in intestinal epithelial cell line [46]. The isoforms IL-32γ, β and δ have been detected in fibroblast-like synoviocytes in rheumatoid arthritis [6]. Among the IL-32 isoforms, Choi *et al.*[4] showed that the IL-32γ is the most active to induce cytokines *in vitro* and *in vivo*. The involvement of IL-32γ in inflammatory disease such as rheumatoid arthritis has been well characterized [12,13,17]. Based on these observations, although different cells in CL or ML lesions can express IL-32 we suggest that in the infiltrate the production of IL-32γ in ATL lesions is mainly due to mononuclear cells and it can contribute to chronic inflammatory process during the disease.

Besides the expression of IL-32, TNF and IL-10 were also investigated in ATL lesions, as these cytokines can be associated [2,7,10-12,15-17]. Similar levels of TNF and IL-10 proteins were detected in CL and ML lesions. In accordance with our results, Faria *et al.*[29] also described similar levels of these proteins in CL and ML lesions. In the present study, in ML and CL lesions there was an increase in TNF gene transcription which is responsible for TNF protein increase. Nevertheless, in ML lesions an accumulation of TNF mRNA is more accentuated than in CL. Several factors control the gene expression at transcriptional and translational levels. The transcription rate is initiated by cell membrane receptor signaling pathways. At this level, we can suggest that parasite intrinsic factors can be different in clones present in CL and in ML lesions leading to a high transcription rate in ML [47]. However, as protein levels were similar in CL and ML in our study, it seems that post-transcriptional mechanisms can be responsible for TNF mRNA accumulation in ML. In macrophages, it has been demonstrated that the increase of secreted TNF protein is largely due to transcriptional mechanism. After cell activation, there is a fast TNF mRNA accumulation and the translation is tightly regulated [48]. These post-transcriptional mechanisms can involve the control of mRNA stability, processing, and translation inhibition [49]. In macrophages activated with lipopolysaccharide, increased expression of HuR, a mRNA binding protein, enhances TNF mRNA stability and reduces translation, causing an accumulation of mRNA and a decrease of TNF secretion [50]. In the current study, ML lesions showed high expression of TNF mRNA that was negatively associated with the levels of TNF protein, suggesting the involvement of inhibitors of TNF mRNA translation, such as HuR. Also microRNAs are induced during inflammation and can control the expression of TNF at translational level. In fibroblast-like synoviocytes from rheumatoid arthritis patients, lipopolysaccharide induces an increase in TNF mRNA without TNF secretion due to miR-346 control [51]. Yet, an unbalance between microRNAs can increase or decrease TNF mRNA translation [52]. Hence, we suggest that in ML mechanisms that control TNF mRNA translation can be more exacerbated than in CL in attempt to decrease TNF production induced by an increased gene transcription. These mechanisms deserve further investigation in CL and ML lesions. We call attention that TNF protein was semi quantified in IHC analyses in this study, and this method cannot have sensitivity enough to detect differences between TNF protein expression in CL and ML lesions.

In this study, IL-10 mRNA levels were similar in CL and ML groups. In previous studies, TNF mRNA levels were showed to be similar between CL and ML patients whereas IL-10 mRNA was higher in ML than in CL lesions, when it was used reverse transcriptase PCR [28,44]. The apparent divergence among these studies may be related to different techniques used to measure mRNA levels or the variability in ATL clinical characteristics of the patients. Here, around 50% of CL patients presented one lesion, lesions were from 0.3 until 8.0 cm in size, and time of lesion evolution in the majority of the patients was ≥ 3 months. In most of ML patients, lesion evolution time was higher than 18 months (two patients presented lesion for 2 and 4 months). It has been described differences in TNF and IL-10 expression between early (<2 - 3 months) and chronic (>4 months) lesions of CL [53,54]. Correlation between size of lesion and TNF production has been reported [33]. In our study, we did not observe any correlation between levels of cytokines (IL-32, TNF, IL-10) and these clinical findings in CL or ML groups. Yet, expression of IL-32 and TNF seems to be independent on the presence or absence of parasites in histopathological exams (data not shown). However, protein levels of IL-10 tend to be higher in fragments of CL lesions that do not present parasites than those positive for parasites (n = 17 positives *vs.* n = 6 negatives, comparing levels of IL-10: p = 0.07). Thus, we suggested that parasites induce immune responses that control the infection and also lead to tissue injury creating a microenvironment that maintain the chronic inflammation. Presence of parasites can be necessary to induce proinflammatory cytokines, but once immune response is established, other factors can induce IL-10 to control the inflammation.

In the present study, analysing correlations between cytokines, positive correlation was found between levels of IL-32γ and TNF proteins in ML but not in CL patients. IL-32 can induce both TNF [2,12] and IL-10 [11,55]. The IL-32-induced TNF production plays a role in inflammatory diseases such as inflammatory bowel disease and rheumatoid arthritis [10,12]. Also, TNF can induce IL-32 creating an auto inflammatory loop [17]. Thus, we suggest that IL-32γ together with TNF, and in the presence of low response to IL-10 in ML [29], can contribute to the maintenance of the chronic inflammation in this clinical form. In CL group it was detected a tendency of positive correlation between TNF and IL-10, what is not observed in ML group, suggesting that in CL the inflammatory mediators can be better balanced than in ML.

In an attempt to characterize the parasites, a PCR reaction was used according to Passos *et al.*[34] and Volpini *et al.*[35]. All positive biopsy fragments from CL and ML lesions presented parasites belonging to subgenus *L*. (*Viannia*). This is the most frequent subgenus of *Leishmania* in South America, specially represented by *L*. (*V.*) *braziliensis,* causing CL or ML [56]. In West Central, North and Northeast of Brazil, >90% of the ATL cases have been reported to be caused by *L. (V.) braziliensis*[57,58]. Only 15.6% of our tested samples were PCR negative, thus in these samples parasites causing ATL could belong to *L*. (*Viannia*) or *L*. (*Leishmania*) subgenus. Because we detected 100% of *L*. (*Viannia*) when PCR was positive, and the high prevalence of *L*. (*V.*) *braziliensis* in Brazil, we investigated the capacity of this species to induce IL-32 in PBMC. Amastigote forms of *L*. (*V.*) *braziliensis* induced IL-32γ mRNA in healthy donor PBMC. In some cell preparations IL-32 was not induced, however in the presence of IFNγ it could be detected (data not shown). The main cell population in PBMC that produces IL-32 seems to be monocytes [5], suggesting that these cells could be the main source of IL-32γ in the inflammatory infiltrates of ATL lesions. Only the IL-32γ isoform was detected in PBMC as well as in ATL lesions, suggesting that the parasites induce only this isoform or even that *L*. (*Viannia*) sp can inhibit the expression of another IL-32 isoforms. These results suggest that in ATL lesions, IL-32 production can be induced by parasites and cytokines such as IFNγ and TNF. Further experiments are needed to understand the mechanisms of IL-32 induction by *Leishmania*. The fact that IL-32 is fast induced in naive PBMC suggests that this cytokine can be involved in infection establishment during the first steps of parasite-host interactions. In this study, the highest IL-32γ mRNA expression level was detected in one lesion of 15 days of evolution. As IL-32 was detected in all lesions evaluated, independent on the evolution time of them, and parasite induced this cytokine in naive PBMC, our results indicate that IL-32 can participate in acute and chronic *Leishmania* infection.

Most of patients studied here were infected with *L*. (*Viannia*) and *L*. (*V*.) *braziliensis* induced IL-32γ in PBMC *in vitro*. This is the first demonstration that *Leishmania* parasites are able to induce IL-32γ and that this cytokine expression can be associated with TNF production in ML. In ATL caused by *L*. (*Viannia*) parasites, it has been demonstrated that proinflammatory Th1 cytokines (TNF, IFNγ) are predominantly expressed in CL and ML lesions [23]. Cellular immune response is more intense in patients infected with *L. (V.) braziliensis* than with *L*. (*L*.) *amazonensis*[23]. In patients with diffuse cutaneous leishmaniasis (DCL) caused by *L. (L.) amazonensis* there is a predominance of IL-10 and Th2 cytokines [59]. In this ATL form, patients do not present cellular immune response [23,60]. Then, ATL can present at the centre of the clinical spectrum localized CL, and two poles - one caused by *L*. (*V*.) *braziliensis* (ML) and the other caused by *L*. (*L*.) *amazonensis* (DCL), with intermediary form between them [59]. Our results indicated that *L*. (*V*.) *braziliensis* induce strong expression of IL-32, suggesting that this cytokine can contribute to proinflammatory immune responses as it does in chronic inflammatory diseases [10,12]. It is possible that in *L*. (*L*.) *amazonensis* infections IL-32 is not highly expressed, especially in DCL. We are investigating this possibility.

Besides the role of IL-32 in chronic inflammatory diseases [10,12] it has been demonstrated that this cytokine can be induced during infections and plays a role in immune response against pathogens [5,8]. In mycobacteria-infected cells IL-32 controls the bacteria growth [20]. Also against viral infections IL-32 plays a role in defence mechanisms [18,19,40]. Based on these observations, a role of IL-32γ in *L. (Viannia) sp* control must be further investigated since this cytokine is present in both CL and ML lesions and is induced by amastigotes in PBMC. This study opens new perspectives on the understanding of immunopathogenesis and control of leishmaniasis caused by New World *Leishmania*.

## Conclusions

This study shows that IL-32 is expressed in lesions of ATL patients. This is the first report about IL-32 in protozoan disease. IL-32 is expressed in several cells present in the leishmaniasis lesions (epithelial and endothelial cells, mononuclear cells, giant cells). The IL-32γ is the unique isoform detected in lesions. No significant differences between IL-32 levels of expression in CL and ML are detected. However, in ML there is a positive correlation between IL-32 and TNF which is not detected in CL. *Leishmania* (*V*.) *braziliensis* is able to induce IL-32γ in PBMC obtained from healthy donors. This study points IL-32 as a target to be investigated in immunopathogenesis and control of ATL.

## Competing interests

Authors declare that there are not any financial competing interests in relation to this manuscript.

## Authors’ contributions

HGJr performed the majority of the experiments helped by AEM, CMG, LLP and FMS. LIPA, FBD and SAP were responsible for diagnosis and follow up of the patients. AKAF and MLD performed the experiments of parasite identification. ACB supervised all immunohistochemical experiments. MMT supervised qPCR. FRD, ACB, LQV and LABJ conceived the experiments and analysed the data. FRD and HGJr wrote de manuscript. MAPO, LQV and LABJ provided suggestions on the manuscript. FRD supervised the research. All authors read and approved the final manuscript.

## Pre-publication history

The pre-publication history for this paper can be accessed here:

http://www.biomedcentral.com/1471-2334/14/249/prepub
